# Editorial: Financial difficulties and mental health problems

**DOI:** 10.3389/fpsyt.2022.1100200

**Published:** 2022-12-01

**Authors:** Thomas Richardson, Sharon Collard, Annie Harper

**Affiliations:** ^1^School of Psychology, School of Geographical Sciences, University of Southampton, Southampton, United Kingdom; ^2^Personal Finance Research Centre, University of Bristol, Bristol, United Kingdom; ^3^School of Medicine, Yale University, New Haven, CT, United States

**Keywords:** finance, financial, debt, mental health, recession, gambling, substance use, homelessness

This special edition, begun in early 2021 had three aims:

- To bridge the gap between academic research, policy and practice.
- To encourage contributions from different disciplines to provide a new range of perspectives and give a bigger picture on the topic.
- To provide an international focus that would balance the dominance of the UK and USA in the existing literature.

We are pleased that our special edition covers an international cohort from Bangladesh, Finland, Sweden, the UK and USA. We also have a range of disciplines and approaches represented within the edition. Although the articles are dominated by health professions including psychiatry, health sciences, addiction, psychology and public health, they do cover both academic and practitioner perspectives. There are also contributions from computer science, social work, economics, mathematics, and social policy as well as input from those with lived experience. The edition showcases a range of methodologies including a case report, national cohort studies, a feasibility study, cross sectional and interview based studies. These are all quantitative, indicating a continued need for qualitative approaches to better understand the lived experience and mechanisms between financial difficulties and mental health. Importantly, the included papers all draw out clear implications for practice and policy of their respective research findings.

A clear theme in the articles is the importance of the macro-economic context. A 20-year follow up study in Finland by Jarroch et al. highlights the impact of a recession on the long-term risk of psychiatric and alcohol related disorders, showing significantly raised risk, but only in men. Two studies examine the impact of the COVID-19 pandemic on mental health in Bangladesh, with a study on wage earners by Sultana et al. showing that those who lost their income had a greater risk of depression and anxiety. Siddique et al. found that depression, anxiety and stress were more common in those with greater risk of unemployment and decreased income. Perhaps unexpectedly, such risk was greater in those with higher family income; this may be explained by the pandemic posing a greater financial risk to those who had more income to lose. These studies show the importance of continuing to monitor and address the financial impacts of the pandemic and recessions, which may be long-term.

Other papers, while acknowledging the significance of the broader context, focus more on understanding individual level processes. Jimenez-Solomon et al. show that for people with psychiatric diagnoses, the impact of objective financial hardship (ability to pay bills, over-indebtedness etc.) on life satisfaction is strongly mediated by subjective financial hardship (dissatisfaction with and shame/stress about finances, and feeling able to plan one's financial futures). The authors suggest that in addition to policies designed to address objective financial hardship, people should be offered shame-resilience skill-building, and peer support. Blair et al. similarly focus on individual level processes, using a case study of one person with bipolar disorder to address the limitations associated with relying on self-reported financial data. They explore the potential for accessing financial data to identify early warning signs and allow for pre-emptive action to prevent further deterioration of a person's financial and mental health, also highlighting associated ethical considerations.

Richardson et al., meanwhile, explore the potential role of technology in addressing the link between finances and mental health. They evaluated a digital CBT intervention – “Space from Money Worries” - designed to address the link between financial difficulties and poor mental health. They find that the intervention is acceptable (positive ratings of modules and a 77% completion rate) with significant impact on symptoms of depression and anxiety, perceived financial wellness and psychological factors in the relationship between financial difficulties and poor mental health.

A third theme in the edition explores the links between financial risk-taking and mental health. The COVID-19 pandemic is likely to have catalyzed an increase in online risky behaviors such as day trading which has a reported association with harmful gambling practices. There is therefore a worrying potential for day trading to cause gambling problems, debt and mental health problems. Such emerging concerns may warrant further research, new screening tools and clinical guidelines. A nationwide register study in Sweden, meanwhile, uses social welfare payment as a proxy to explore economic hardship as a potential risk factor that might contribute to intentional self-harm in people with gambling disorder (Karlsson et al.). While the study does not find social welfare payments to be a significant risk factor for intentional self-harm among this population, nonetheless the findings indicate that social services departments should pay greater attention to suicidality and self-injurious behavior among recipients of social welfare payment given the prevalence of intentional self-harm among this group.

Finally, a review paper by Maguire discusses the different types of debt seen in homelessness including rent, health and drug debt, and how they maintain homelessness. This review considers possible interventions in terms of budgeting, systemic organizational change and psychological interventions such as motivational interviewing.

This special edition shines a spotlight on the complex links and interactions between financial difficulties and mental health problems, from different disciplinary perspectives and utilizing a range of research methods. It has achieved its aim of putting this topic more firmly on the academic radar but there is clearly great potential for further work as we continue to face global economic turbulence with record inflation and a cost of living crisis in many countries. We would like to thank all of the authors who contributed and the participants who took part to make this special edition possible.

## Author contributions

All authors listed have made a substantial, direct, and intellectual contribution to the work and approved it for publication.

## Conflict of interest

Author TR received payment for developing, and receives royalties from use of Space from Money Worries which is discussed in this editorial. Author SC receives funding from GambleAware, a national charity operating in Great Britain. The remaining author declares that the research was conducted in the absence of any commercial or financial relationships that could be construed as a potential conflict of interest.

## Publisher's note

All claims expressed in this article are solely those of the authors and do not necessarily represent those of their affiliated organizations, or those of the publisher, the editors and the reviewers. Any product that may be evaluated in this article, or claim that may be made by its manufacturer, is not guaranteed or endorsed by the publisher.

